# Marine water environmental DNA metabarcoding provides a comprehensive fish diversity assessment and reveals spatial patterns in a large oceanic area

**DOI:** 10.1002/ece3.6482

**Published:** 2020-06-23

**Authors:** Natalia Fraija‐Fernández, Marie‐Catherine Bouquieaux, Anaïs Rey, Iñaki Mendibil, Unai Cotano, Xabier Irigoien, María Santos, Naiara Rodríguez‐Ezpeleta

**Affiliations:** ^1^ AZTI, Marine Research Basque Research and Technology Alliance (BRTA) Sukarrieta Spain; ^2^ AZTI, Marine Research Basque Research and Technology Alliance (BRTA) Pasaia Spain; ^3^ IKERBASQUE Basque Foundation for Science Bilbao Spain

**Keywords:** Actinopterygii, Elasmobranchii, environmental DNA, marine fish surveys, metabarcoding

## Abstract

Current methods for monitoring marine fish (including bony fishes and elasmobranchs) diversity mostly rely on trawling surveys, which are invasive, costly, and time‐consuming. Moreover, these methods are selective, targeting a subset of species at the time, and can be inaccessible to certain areas. Here, we used environmental DNA (eDNA), the DNA present in the water column as part of shed cells, tissues, or mucus, to provide comprehensive information about fish diversity in a large marine area. Further, eDNA results were compared to the fish diversity obtained in pelagic trawls. A total of 44 5 L‐water samples were collected onboard a wide‐scale oceanographic survey covering about 120,000 square kilometers in Northeast Atlantic Ocean. A short region of the 12S rRNA gene was amplified and sequenced through metabarcoding generating almost 3.5 million quality‐filtered reads. Trawl and eDNA samples resulted in the same most abundant species (European anchovy, European pilchard, Atlantic mackerel, and blue whiting), but eDNA metabarcoding resulted in more detected bony fish and elasmobranch species (116) than trawling (16). Although an overall correlation between fishes biomass and number of reads was observed, some species deviated from the common trend, which could be explained by inherent biases of each of the methods. Species distribution patterns inferred from eDNA metabarcoding data coincided with current ecological knowledge of the species, suggesting that eDNA has the potential to draw sound ecological conclusions that can contribute to fish surveillance programs. Our results support eDNA metabarcoding for broad‐scale marine fish diversity monitoring in the context of Directives such as the Common Fisheries Policy or the Marine Strategy Framework Directive.

## Graphical abstract

eDNA samples provide information on fish diversity in a broad‐scale marine area, detecting almost ten times more fish species compared with pelagic trawling, including some considered elusive or difficult to capture with traditional fishing methods. The potential of eDNA is particularly relevant in a context of global change, where establishing efficient management actions based on numerous, continuous, and accurate biodiversity assessments is paramount.

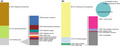

## 1 INTRODUCTION

Monitoring of marine biodiversity provides a baseline for policy implementation toward a sustainable use of the marine environment and its resources. Among the traditional methods for surveying marine fauna, trawling has been widely used, as identification and quantification of large volumes of organisms are considered a reliable method for monitoring bony fishes and elasmobranchs (hereafter fishes) and other marine animal populations (ICES, 2015; Massé, Uriarte, Angélico, & Carrera, 2018). Fish surveys using trawls are conditioned by the gear's own characteristics (e.g., mesh size, area of opening) and deployment parameters (e.g., towing speed, depth, and diel variation) (Heino et al., 2011). Consequently, besides being invasive and time‐consuming, fish trawling in pelagic environments can be largely selective affecting diversity estimates and knowledge of species composition (Fraser, Greenstreet, & Piet, 2007; ICES, 2004). For instance, due to their large body size, fast swimming speed, and in some cases, scarcity, many elasmobranch species are not thoroughly surveyed (Rago, 2004). Therefore, alternative methods are needed, and advances in DNA sequencing and bioinformatics have opened new avenues to assess marine biodiversity in a noninvasive manner (Danovaro et al., 2016; Rees, Maddison, Middleditch, Patmore, & Gough, 2014).

In particular, the analysis of environmental DNA (eDNA), that is, the genetic material shed and excreted by organisms to the environment, to characterize the biological communities present in an environment (Taberlet, Coissac, Hajibabaei, & Rieseberg, 2012) is gaining increasing attention for monitoring aquatic environments (Thomsen & Willerslev, 2015). Community composition can be inferred from eDNA samples through metabarcoding, whereby the eDNA is collected from the water column through filtering, selectively amplified through PCR using primers targeting a given barcode from a particular taxonomic group and sequenced (Taberlet, Coissac, Pompanon, Brochmann, & Willerslev, 2012). The resulting sequences are then compared against a reference database to perform biodiversity inventories (Deiner, Bik, & Mächler, 2017). Besides the inherent biases of metabarcoding (Aylagas, Borja, Irigoien, & Rodríguez‐Ezpeleta, 2016), the use of eDNA adds additional biases due to the complex ecology of this molecule (Barnes & Turner, 2016) that might interfere with its potential use for biodiversity assessment. Thus, additional research is required to better understand the utility of eDNA for fish monitoring. Most studies using eDNA metabarcoding for monitoring fish communities are based on freshwater environments and have shown that eDNA metabarcoding provides overall estimates that are equivalent or superior to traditional methods such as visual surveys, trawling, or electrofishing (Hänfling et al., 2016; Minamoto, Yamanaka, Takahara, Honjo, & Zi, 2012; Pont et al., 2018).

As opposed to freshwater systems, the marine environment has in general a larger water volume to fish biomass ratio and is influenced by currents, implying that the eDNA is less concentrated and disperses quicker (Hansen, Bekkevold, Clausen, & Nielsen, 2018). This, coupled with a higher sympatric marine fish diversity, suggests that monitoring fish diversity through eDNA sampling could be particularly challenging in the marine environment. Indeed, only a handful of studies have applied eDNA metabarcoding for monitoring fish in natural marine environments (e.g., O’Donnell et al., 2017; Stat et al., 2017). Among them, only a few have compared eDNA and other traditional surveying methods and are based on a very small area of a few square kilometers either in ports (Jeunen et al., 2019; Sigsgaard et al., 2017; Thomsen et al., 2012) or in coastal areas (Andruszkiewicz et al., 2017; DiBattista et al., 2017; Yamamoto et al., 2017) or have performed comparisons at family level taxonomic assignments (Thomsen et al., 2016). Thus, although these studies envision eDNA metabarcoding as a promising method for noninvasive, faster, more efficient, and reliable marine surveys, this needs still to be tested in the context of a fishery survey covering a broad marine area.

The Bay of Biscay is a biogeographical area in the North Atlantic Region covering more than 220,000 km^2^, at which the main economic activities include commercial fishing. Large populations of species such as the European anchovy *Engraulis encrasicolus*, the European pilchard *Sardina pilchardus*, the European hake *Merluccius merluccius*, the Atlantic Mackerel *Scomber scombrus*, and the Atlantic horse mackerel *Trachurus trachurus* are dominant in the area (ICES, 2018). Fish diversity in the Bay of Biscay has been accounted using mainly observational methods, fish trawling, and acoustic surveys; thus, there is scope for incorporating and assessing the performance of eDNA‐based surveys. This paper aims to test the potential of eDNA metabarcoding to assess the fish community composition in a large marine area, such as the Bay of Biscay. For that aim, we have compared eDNA metabarcoding‐based biodiversity estimates with those derived from fishing trawls catches and have related eDNA metabarcoding‐based estimates with the known spatial distribution and ecological patterns of the species in the area.

## 2 METHODS

### 2.1 Sample collection

**FIGURE 1:**
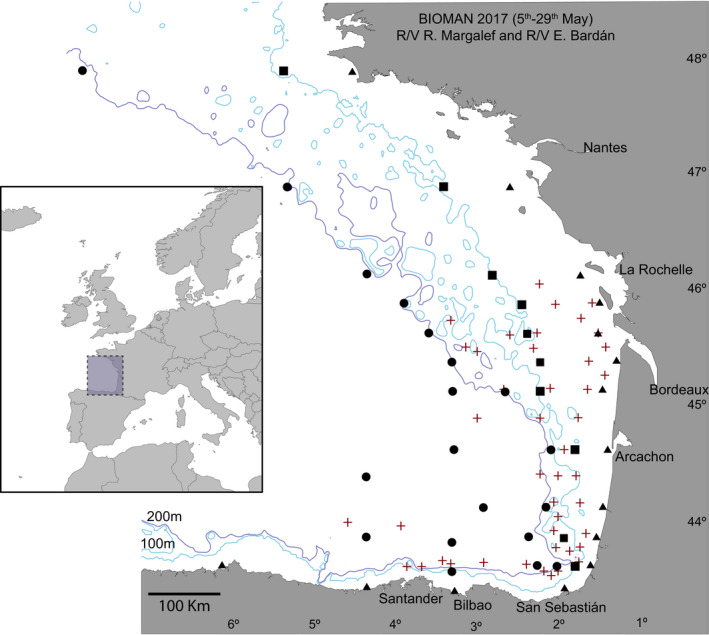
Study area and sampling sites for the BIOMAN 2017 survey in the Bay of Biscay. Triangles represent eDNA sampling sites where station depth was <90 m, squares, eDNA sampling sites with depths between 90 and 127 m, and circles, eDNA sampling sites with >127 m depths. Crosses are located where pelagic fishing trawls were deployed. 100 m and 200 m isobaths are shown

Fish and elasmobranchs catches and water samples were collected during the BIOMAN 2017 survey (Santos, Ibaibarriaga, Louzao, Korta, & Uriarte, 2018) between May 5 and May 29, 2017, covering the area of about 120,000 km^2^ between the French continental shelf and the Spanish shelf (Figure 1) on board the Emma Bardán and Ramón Margalef research vessels. Fish catches were obtained on board the R/V Emma Bardán pelagic trawler. The trawl had an 8 mm mesh size cod end, and towing time and speed were 40 min and 4 knots, respectively. A total of 44 stations were used for trawling. Although station depths varied between 26 and 3,000 m, the maximum fishing depth was 156 m. Onboard, fish were morphologically identified to species level or, when doubt, to the smallest taxonomic rank (e.g., family or genus). Biomass estimates were standardized as Kg caught per taxa and per station. In 44 additional stations (Figure 1), water samples were collected on board the R/V Ramón Margalef research vessel using the continuous circuit intake of the ship at 4.4 m depth, transferred to 5‐L plastic bottles and filtered through Sterivex 0.45 µm pore size enclosed filters (Millipore) with a peristaltic pump, using a 6 μm mesh size net in the incoming tube to avoid clogging. All material used for filtering, including tubes, net, and bottles were decontaminated by rinsing them once with 10% bleach solution, three times with Milli‐Q water and three times with the sampling water to be filtered. Filters were kept at −20°C until further processing.

### 2.2 DNA extraction and amplicon library preparation

DNA extractions were performed in a dedicated pre‐PCR laboratory using the DNeasy^®^ blood and tissue kit (Qiagen) following the modified protocol for DNA extraction from Sterivex filters without preservation buffer by Spens, Evans, and Halfmaerten (2017). DNA concentration was measured with the Quant‐iT dsDNA HS assay kit using a Qubit^®^ 2.0 Fluorometer (Life Technologies, California, USA). DNA from all 44 samples was amplified with the teleo_F/telo_R primer pair (hereafter “teleo”), targeting a region (~60 bp) of the mitochondrial 12S rRNA gene, combined with the human blocking primer teleo_blk (Valentini et al., 2016). PCR mixtures were prepared under the hood in the pre‐PCR laboratory using dedicated micropipettes and disposable plastic ware that were previously decontaminated under the UV light, and all postamplification steps were carried out in the post‐PCR laboratory. Three replicate PCR amplifications were done per sample in a final volume of 20 µl including 10 µl of 2X Phusion Master Mix (Thermo Scientific, Massachusetts, USA), 0.4 µl of each amplification primer (final concentration of 0.2 µM), 4 µl of teleo_blk (final concentration of 2 µM), 3.2 µl of Milli‐Q water, and 2 µl of 10 ng/µl template DNA. Samples from 4 stations were also amplified (a) using the same procedure but without the blocking primer, and (b) using the mlCOIintF/dgHCO2198 primer pair (hereafter “mlCOI”), targeting a region (⁓310 bp) of the COI gene (Leray et al., 2013; Meyer, 2003). The thermocycling profile for PCR amplification included 3 min at 98°C; 40 or 35 cycles (for “teleo” and “mlCOI” as indicated in Valentini et al. (2016) and Leray et al. (2013), respectively) of 10 s at 98°C, 30 s at 55, or 46°C (for “teleo” and “mlCOI,” respectively) and 45 s at 72°C, and finally, 10 min at 72°C. Replicate PCR products were combined and purified using AMPure XP beads (Beckman Coulter, California, USA) following manufacturer's instructions and used as templates for the generation of 12 × 8 dual‐indexed amplicons in the second PCR following the “16S Metagenomic Sequence Library Preparation” protocol (Illumina, California, USA) using the Nextera XT Index Kit (Illumina, California, USA). PCR negative controls resulted in no visible amplification in agarose gels. Multiplexed PCR products were purified using the AMPure XP beads, quantified using Quant‐iT dsDNA HS assay kit using a Qubit^®^ 2.0 Fluorometer (Life Technologies, California, USA), and adjusted to 4 nM. Five microlitre of each sample were pooled, checked for size and concentration using the Agilent 2100 bioanalyzer (Agilent Technologies, California, USA), sequenced using the 2 × 300 paired end protocol on the Illumina MiSeq platform (Illumina, California, USA), and demultiplexed based on their barcode sequences.

### 2.3 Reference database

Two reference databases were created for the “teleo” barcode. A first “global” database included all Chordata 12S rRNA and complete mitochondrial genome sequences available from GenBank (accessed in February 2018). By performing an all‐against‐all BLAST (Altschul, Gish, Miller, Myers, & Lipman, 1990), potential sources of contamination or erroneous taxonomic assignments were removed such as human contaminations (e.g., nonhuman labeled sequences that matched at 100% identity with the *Homo sapiens* 12S rRNA sequence) or cross‐contaminated sequences (e.g., sequences arising from the same study that, even when belonging to different genus, were 100% identical). All sequences were trimmed to the “teleo” region. Taxonomy for the GenBank sequences was retrieved using E‐utilities (Sayers, 2008) and modified to match that of the World Register of Marine Species: WoRMS (Horton, Kroh, & Ahyong, 2018), forcing for seven taxonomic levels, that is, Phylum, Subphylum, Class, Order, Family, Genus, and Species. This “global” reference database contains 10,284 “teleo” region sequences. For the second database, only sequences from target species were retrieved so that more exhaustive error checking was possible. The list of the 1,858 fish species expected in the Northeast Atlantic and Mediterranean areas was compiled from FishBase (http://www.fishbase.org), and their corresponding scientific names and sequences were obtained from NCBI (https://www.ncbi.nlm.nih.gov). For the retrieved records, only those covering the “teleo” region were selected and aligned. A phylogenetic tree was built with RAxML (Stamatakis, 2014) using the GTR‐CAT model and visualized with iTOl (Letunic & Bork, 2016). The tree was visually inspected, and the records corresponding to misplaced species were removed from the database. This “local” reference database contains “teleo” region sequences of 612 species. For the “mlCOI” barcode, the reference database consisted in the COI sequences and their corresponding taxonomy obtained from the BOLD (Ratnasingham & Hebert, 2007) database.

### 2.4 Read preprocessing, clustering, and taxonomic assignment

Overall quality of raw demultiplexed reads was verified with *FASTQC* (Andrews, 2010). Forward and reverse primers were removed with *cutadapt* (Martin, 2011) allowing a maximum error rate of 20%, discarding read pairs that do not contain the two primer sequences and retaining only those reads longer than 30 nucleotides. Paired reads were merged using *pear* (Zhang, Kobert, Flouri, & Stamatakis, 2014) with a minimum overlap of 20 nucleotides. Pairs with average quality lower than 25 Phred score were removed using *Trimmomatic* (Bolger, Lohse, & Usadel, 2014). *mothur* (Schloss et al., 2009) was used to remove reads (a) not covering the target region, (b) shorter than 40 or 313 nucleotides, for “teleo” and “mlCOI,” respectively, (c) containing ambiguous positions, and (d) being potential chimeras, which were detected based on the *UCHIME* algorithm (Edgar, Haas, Clemente, Quince, & Knight, 2011). Reads were clustered into OTUs using *vsearch* (Rognes, Flouri, Nichols, Quince, & Mahé, 2016) at 97% similarity threshold or using *Swarm* (Mahé, Rognes, Quince, de Vargas, & Dunthorn, 2014) with a d value of 1. In both cases, the *LULU* postclustering algorithm (Frøslev et al., 2017) was applied with a minimum threshold of sequence similarity for considering any OTU as an error of 97%. Taxonomic assignment of unique reads and of representative sequences for each OTU was performed using the naïve Bayesian classifier method (Wang, Garrity, Tiedje, & Cole, 2007) implemented in *mothur* using the 12S rRNA and COI databases described above. Reads with the same taxonomic assignment were grouped into phylotypes.

### 2.5 Biodiversity analyses

Analyses were performed in R v3.6.1 with the packages *Phyloseq* v1.22.3 (McMurdie & Holmes, 2013) and *Vegan* v2.5‐6 (Oksanen, Blanchet, & Friendly, 2019). Sampling stations were classified into three categories considering their depth (see Map in Figure 1) and grouped so that samples around the 100 isobath are grouped together: shallow stations where maximum station depth was <90 m, medium stations, when depth ranged between 90 and 127 m, and deep stations where depth was >127 m. To assess differences in fish diversity across categories (i.e., according to shallow, medium, and deep stations), we calculated the Bray–Curtis dissimilarity index for relative abundance of species with the function *ordinate* using only phylotypes with more than 10 reads. These distances were then ordinated using a nonmetric Multidimensional Scaling (NMDS) as implemented in *Phyloseq* and differences between stations were tested with PERMANOVA (1,000 permutations) using the function *adonis* within the R package *Vegan* previous testing for homogeneity of variance using the function *betadisper*. A linear model was used on species with more than 1,000 reads, to test for the effect of the abundance of reads (previously standardized according to the overall number of reads and stations per zone), and the distance from the coast. An overall correlation between the log‐transformed values (to deal with high variation on the relative scale) of the number of reads obtained and the biomass caught per species was explored with the Pearson correlation coefficient, using a *t* test to establish whether the correlation coefficient is significantly different from zero, as implemented in R package *Stats* v0.1.0. For an even geographic distribution between water and fish sampling sites, a total of nine water sampling sites north La Rochelle were removed for the comparison analyses. In addition, in order to compare eDNA and trawling‐based estimates at a smaller scale, we created groups of stations so that this comparison was possible. For that aim, we combined the data from all eDNA and trawling stations within <20 nautical miles of each eDNA station in what we call mega‐stations. A total of 30 mega‐stations resulted. A Mantel test as implemented in the R package *ade4* v1.7‐13 (Dray & Dufour, 2007) was used to explore correlation between the mega‐station geographic and Bray–Curtis distance matrices of. The bias‐corrected Chao II species richness was estimated as in Olds et al. (2016). The list of species commonly reported from the Bay of Biscay was obtained mainly from (a) Basterretxea, Oyarzabal, and Artetxe (2012), (b) the AZTI’s database on fish bottom trawling discards in the area gathered according to EU regulation 2017/1004 of 17 May 2017, (c) the data obtained from fish pelagic trawling during BIOMAN surveys from 2003 until 2019, (d) the ICES database for International Bottom Trawling Surveys available from www.ices.dk, and (e) the 2017 Pélagiques Gascogne (PELGAS) integrated survey (Mathieu, Laurence, & Patrick, 2019).

## 3 RESULTS

### 3.1 Data quality and overall taxonomic composition

We obtained a total of 4,640,913 raw “teleo” reads from which 3,366,264 (72%) were retained after quality check for downstream analyses. The average number of “teleo” reads per sample was 70,131 (Table 1). Using the “global” database, 99.88% of the reads were classified as Actinopterygii or Elasmobranchii. The remaining were classified as mammals (40.16%) and birds (9.60%), with half of the reads (50.24%) not classified into Class level. Only 14 reads in eight samples were specifically assigned to *H. sapiens*. From these, two samples did not include the specific blocking primer used, suggesting that samples held very little contamination from external sources. Using the “local” database, 99.98% of the reads were classified either as Actinopterygii or Elasmobranchii and, depending on the clustering method used, the number of taxa recovered varied. *swarm* clustering yielded 90 OTUs identified at the species level (including 95.5% of the reads) and *vsearch*, 109 (including 95% of the reads), whereas not clustering reads into OTUs, but using phylotypes, resulted in 116 Actinopterygii and Elasmobranchii species (including 95% of the reads) identified. Further analyses were based on phylotypes assigned to the species level (Table 2) as no additional information is provided by using OTU clustered reads. From the 116 identified species, 50 included more than 10 reads.

**TABLE 1:** Station depth, category, and number of reads obtained per sample after sequencing, removing primers, pair‐assembling, quality filtering, primer mapping, and chimera removal for the *teleo* region

Sample	Station depth (m)	Category	Raw	Retained after primer checking	Retained after merging	Retained after quality filtering	Retained after mapping to teleo region	Retained after chimera removal	% of retained reads for analysis
Sample_01	27	Shallow	127,549	100,839	99,036	99,036	95,240	95,240	74.67
Sample_02	1,315	Deep	99,724	96,995	90,080	90,080	89,206	89,206	89.45
Sample_03	764	Deep	67,867	49,789	39,229	39,229	28,896	28,896	42.58
Sample_04	46	Shallow	93,699	89,918	85,894	85,894	83,153	83,153	88.74
Sample_05	43	Shallow	157,845	150,348	145,987	145,987	100,388	100,387	63.60
Sample_06	180	Deep	120,961	116,014	103,982	103,982	101,419	101,418	83.84
Sample_07	508	Deep	55,396	36,121	33,605	33,605	16,062	16,061	28.99
Sample_08	1,373	Deep	104,158	77,717	71,716	71,716	65,091	65,091	62.49
Sample_13	91	Medium	138,472	134,943	122,420	122,420	117,565	117,562	84.90
Sample_14	735	Deep	66,247	50,282	21,624	21,624	18,813	18,813	28.40
Sample_15	639	Deep	98,224	96,676	91,079	91,079	89,581	89,581	91.20
Sample_16	25	Shallow	94,195	92,482	92,074	92,074	87,100	87,100	92.47
Sample_17	741	Deep	60,308	38,492	24,125	24,125	20,355	20,355	33.75
Sample_18	127	Medium	101,688	99,550	98,456	98,456	97,918	97,918	96.29
Sample_19	38	Shallow	119,881	113,505	104,320	104,320	99,181	99,181	82.73
Sample_20	1,285	Deep	111,757	107,998	103,660	103,660	96,993	96,993	86.79
Sample_21	300	Deep	134,490	132,496	130,290	130,290	121,976	121,976	90.70
Sample_22	33	Shallow	88,044	78,156	50,143	50,143	43,798	43,798	49.75
Sample_23	968	Deep	52,240	39,687	16,584	16,584	12,090	12,090	23.14
Sample_24	169	Deep	104,423	97,858	77,320	77,320	65,788	65,788	63.00
Sample_25	23	Shallow	89,199	79,999	68,124	68,124	59,414	59,414	66.61
Sample_26	132	Deep	110,206	106,817	106,547	106,547	105,436	105,436	95.67
Sample_27	1,003	Deep	99,856	94,244	75,172	75,172	69,577	69,577	69.68
Sample_28	112	Medium	100,002	98,722	97,656	97,656	97,462	97,462	97.46
Sample_29	38	Shallow	87,452	73,824	61,161	61,161	51,590	51,590	58.99
Sample_30	24	Shallow	155,459	153,556	153,517	153,517	152,877	152,877	98.34
Sample_31	100	Medium	91,723	77,592	65,748	65,748	57,283	57,283	62.45
Sample_32	185	Deep	76,396	64,566	52,863	52,863	35,175	35,175	46.04
Sample_33	480	Deep	81,771	74,171	59,470	59,470	54,788	54,788	67.00
Sample_34	104	Medium	70,037	56,967	54,193	54,193	45,712	45,712	65.27
Sample_35	28	Shallow	124,438	122,670	122,076	122,076	113,812	113,812	91.46
Sample_36	25	Shallow	108,071	107,183	107,166	107,166	106,898	106,898	98.91
Sample_37	96	Medium	138,090	134,214	132,943	132,943	131,644	131,644	95.33
Sample_38	590	Deep	89,791	77,675	72,468	72,468	59,562	59,562	66.33
Sample_39	1,010	Deep	38,021	26,356	21,918	21,918	16,839	16,839	44.29
Sample_40	108	Medium	79,924	74,147	67,941	67,941	64,152	64,152	80.27
Sample_41	26	Shallow	119,488	117,958	117,881	117,881	116,414	116,414	97.43
Sample_42	30	Shallow	135,958	133,840	46,726	46,726	46,640	46,640	34.30
Sample_43	104	Medium	115,584	109,058	76,209	76,209	71,558	71,558	61.91
Sample_44	185	Deep	25,519	24,976	8,064	8,064	7,755	7,755	30.39
Sample_45	33	Shallow	115,109	113,582	99,454	99,454	93,219	93,219	80.98
Sample_46	90	Medium	121,100	119,555	119,036	119,036	106,640	106,636	88.06
Sample_47	675	Deep	68,697	55,896	19,019	19,019	6,716	6,716	9.78
Sample_48	110	Medium	94,499	91,697	90,715	90,715	89,305	89,305	94.50
Sample_01NOBP	27	Shallow	115,003	97,454	93,987	93,987	82,749	82,748	71.95
Sample_27NOBP	1,003	Deep	88,793	78,114	51,755	51,755	43,902	43,902	49.44
Sample_32NOBP	185	Deep	57,276	38,142	29,015	29,015	26,686	26,686	46.59
Sample_47NOBP	675	Deep	46,283	35,228	7,304	7,304	1857	1857	4.01
TOTAL			4,333,558	3,989,131	3,497,691	3,497,691	3,211,081	3,211,071	
AVERAGE_all			98,489.95	90,662.07	79,492.98	79,492.98	72,979.11	72,978.89	69.52

**TABLE 2:** Number of reads, relative abundance, and taxonomic information recovered from eDNA by the 12S rRNA mitochondrial marker in the Bay of Biscay during the BIOMAN 2017 survey

Number of reads	Relative abundance (%)	Class	Family	Species
1,791,393	51.67	Actinopterygii	Engraulidae	*Engraulis encrasicolus*
959,248	27.67	Actinopterygii	Clupeidae	*Sardina pilchardus*
172,116	4.96	Actinopterygii	Scombridae	*Scomber scombrus*
119,672	3.45	Actinopterygii	unclassified	unclassified
81,658	2.36	Actinopterygii	Gadidae	*Micromesistius poutassou*
52,853	1.52	Actinopterygii	Sparidae	*Diplodus sargus*
41,467	1.20	Actinopterygii	Sparidae	*Pagellus acarne*
29,792	0.86	Actinopterygii	Molidae	*Mola mola*
25,536	0.74	Actinopterygii	Moronidae	*Dicentrarchus labrax*
22,982	0.66	Actinopterygii	Lophiidae	*Lophius piscatorius*
17,875	0.52	Actinopterygii	Mugilidae	*Chelon ramada*
17,307	0.50	Actinopterygii	Scombridae	unclassified
16,971	0.49	Actinopterygii	unclassified	unclassified
16,859	0.49	Actinopterygii	Ammodytidae	*Ammodytes dubius*
14,161	0.41	Actinopterygii	Gobiidae	*Gobius niger*
11,677	0.34	Actinopterygii	Labridae	*Ctenolabrus rupestris*
10,024	0.29	Actinopterygii	Gobiidae	unclassified
8,912	0.26	Actinopterygii	Argentinidae	*Argentina silus*
7,331	0.21	Elasmobranchii	Somniosidae	*Somniosus microcephalus*
7,158	0.21	Actinopterygii	Gobiidae	*Buenia affinis*
4,464	0.13	Actinopterygii	Scombridae	*Scomber colias*
4,456	0.13	Actinopterygii	Merlucciidae	*Merluccius merluccius*
3,577	0.10	Actinopterygii	Clupeidae	*Alosa fallax*
3,128	0.09	Actinopterygii	Mugilidae	*Chelon aurata*
2,527	0.07	Actinopterygii	Sparidae	*Pagellus bogaraveo*
2078	0.06	Actinopterygii	Labridae	*Labrus merula*
2075	0.06	Actinopterygii	Cyprinidae	unclassified
1921	0.06	Actinopterygii	Alepocephalidae	*Xenodermichthys copei*
1,284	0.04	Elasmobranchii	Carcharhinidae	*Prionace glauca*
1,284	0.04	Actinopterygii	Cyprinidae	*Rutilus rutilus*
1,249	0.04	Actinopterygii	Labridae	*Coris julis*
1,189	0.03	Actinopterygii	Myctophidae	unclassified
1,096	0.03	Actinopterygii	Sparidae	unclassified
996	0.03	Actinopterygii	Soleidae	*Microchirus azevia*
989	0.03	Actinopterygii	Bathylagidae	*Bathylagus euryops*
986	0.03	Actinopterygii	Cyprinidae	*Blicca bjoerkna*
971	0.03	Actinopterygii	Scombridae	*Katsuwonus pelamis*
806	0.02	Actinopterygii	Clupeidae	unclassified
695	0.02	Actinopterygii	Labridae	*Symphodus melops*
654	0.02	Actinopterygii	unclassified	unclassified
653	0.02	Actinopterygii	Cyprinidae	unclassified
591	0.02	Actinopterygii	Soleidae	*Solea solea*
570	0.02	unclassified	unclassified	unclassified
527	0.02	Actinopterygii	Clupeidae	*Alosa alosa*
384	0.01	Actinopterygii	Sparidae	unclassified
350	0.01	Elasmobranchii	Rajidae	*Raja undulata*
338	0.01	Actinopterygii	Gadidae	unclassified
299	0.01	Actinopterygii	Mugilidae	*Chelon labrosus*
188	0.01	Actinopterygii	Sparidae	*Pagrus major*
167	0.00	Actinopterygii	Scombridae	unclassified
163	0.00	Actinopterygii	Trachinidae	*Trachinus draco*
70	0.00	Actinopterygii	Scombridae	unclassified
64	0.00	Elasmobranchii	unclassified	unclassified
62	0.00	Elasmobranchii	Lamnidae	*Lamna nasus*
57	0.00	Actinopterygii	unclassified	unclassified
53	0.00	Actinopterygii	Gadidae	*Gadus morhua*
50	0.00	Actinopterygii	Gobiidae	unclassified
46	0.00	Actinopterygii	Gadidae	*Gadiculus thori*
43	0.00	Elasmobranchii	unclassified	unclassified
35	0.00	Actinopterygii	Gobiidae	*Neogobius melanostomus*
34	0.00	Actinopterygii	Carangidae	*Trachurus trachurus*
29	0.00	Actinopterygii	Myctophidae	*Notoscopelus kroyeri*
28	0.00	Actinopterygii	Sparidae	*Stenotomus chrysops*
25	0.00	Actinopterygii	Labridae	unclassified
25	0.00	Actinopterygii	Myctophidae	unclassified
21	0.00	Actinopterygii	Cyprinidae	*Squalius cephalus*
19	0.00	Actinopterygii	Clupeidae	unclassified
17	0.00	Actinopterygii	Myctophidae	*Benthosema glaciale*
16	0.00	Actinopterygii	Scombridae	*Scomber australasicus*
13	0.00	Actinopterygii	Gempylidae	*Gempylus serpens*
13	0.00	Actinopterygii	Scombridae	*Thunnus orientalis*
12	0.00	Actinopterygii	unclassified	unclassified
11	0.00	Actinopterygii	Eurypharyngidae	*Eurypharynx pelecanoides*
10	0.00	Elasmobranchii	unclassified	unclassified
9	0.00	Actinopterygii	Labridae	*Tautogolabrus adspersus*
9	0.00	Actinopterygii	Lotidae	*Ciliata mustela*
8	0.00	Actinopterygii	Carangidae	unclassified
8	0.00	Actinopterygii	unclassified	unclassified
8	0.00	Actinopterygii	Gempylidae	unclassified
8	0.00	Actinopterygii	Soleidae	unclassified
8	0.00	Actinopterygii	Pomacentridae	*Abudefduf saxatilis*
8	0.00	Actinopterygii	Clupeidae	*Alosa sapidissima*
8	0.00	Actinopterygii	Myctophidae	*Lampanyctus crocodilus*
7	0.00	Actinopterygii	unclassified	unclassified
7	0.00	Elasmobranchii	Glaucostegidae	*Glaucostegus cemiculus*
7	0.00	Actinopterygii	unclassified	unclassified
7	0.00	Actinopterygii	Gobiidae	*Odondebuenia balearica*
7	0.00	Actinopterygii	Sparidae	unclassified
7	0.00	Actinopterygii	Sparidae	*Sparus aurata*
6	0.00	Actinopterygii	Labridae	*Symphodus cinereus*
6	0.00	Actinopterygii	Mugilidae	unclassified
6	0.00	Elasmobranchii	Somniosidae	unclassified
6	0.00	Actinopterygii	Nettastomatidae	unclassified
6	0.00	Actinopterygii	Alepocephalidae	unclassified
6	0.00	Actinopterygii	Scombridae	*Acanthocybium solandri*
5	0.00	Actinopterygii	Sparidae	*Pagellus erythrinus*
5	0.00	Actinopterygii	Pomacentridae	unclassified
5	0.00	Actinopterygii	Gobiidae	*Thorogobius ephippiatus*
5	0.00	Actinopterygii	Scombridae	*Thunnus obesus*
5	0.00	Actinopterygii	Gadidae	*Trisopterus minutus*
4	0.00	Actinopterygii	Molidae	unclassified
4	0.00	Actinopterygii	Labridae	*Bodianus speciosus*
4	0.00	Actinopterygii	Gadidae	*Merlangius merlangus*
4	0.00	Actinopterygii	Mugilidae	*Mugil bananensis*
4	0.00	Actinopterygii	Moronidae	*Dicentrarchus punctatus*
4	0.00	Actinopterygii	unclassified	unclassified
4	0.00	Actinopterygii	Gempylidae	*Nealotus tripes*
4	0.00	unclassified	unclassified	unclassified
3	0.00	Actinopterygii	Paralepididae	*Magnisudis atlantica*
3	0.00	Actinopterygii	Macrouridae	unclassified
3	0.00	Actinopterygii	Cyprinidae	*Leuciscus idus*
3	0.00	Actinopterygii	Derichthyidae	unclassified
3	0.00	Actinopterygii	Scombridae	*Auxis thazard*
3	0.00	Actinopterygii	Gonostomatidae	*Sigmops bathyphilus*
3	0.00	Actinopterygii	Macrouridae	unclassified
2	0.00	Actinopterygii	Molidae	*Ranzania laevis*
2	0.00	Actinopterygii	Lutjanidae	*Lutjanus argentimaculatus*
2	0.00	Actinopterygii	Scombridae	*Euthynnus alletteratus*
2	0.00	Actinopterygii	Gonostomatidae	unclassified
2	0.00	Actinopterygii	Carangidae	*Alectis ciliaris*
2	0.00	Actinopterygii	Syngnathidae	unclassified
2	0.00	Actinopterygii	Molidae	*Masturus lanceolatus*
2	0.00	Actinopterygii	Labridae	unclassified
2	0.00	Actinopterygii	Mugilidae	unclassified
2	0.00	Actinopterygii	Liparidae	*Paraliparis copei copei*
2	0.00	Actinopterygii	Myctophidae	*Lampanyctus macdonaldi*
2	0.00	Actinopterygii	unclassified	unclassified
2	0.00	Actinopterygii	Luvaridae	*Luvarus imperialis*
2	0.00	Actinopterygii	Clupeidae	*Brevoortia tyrannus*
2	0.00	Elasmobranchii	Dalatiidae	*Dalatias licha*
2	0.00	Elasmobranchii	Carcharhinidae	unclassified
2	0.00	Actinopterygii	Cyprinidae	*Phoxinus ujmonensis*
2	0.00	Actinopterygii	Gempylidae	*Diplospinus multistriatus*
2	0.00	Actinopterygii	Echeneidae	unclassified
1	0.00	Actinopterygii	Pomacentridae	unclassified
1	0.00	Actinopterygii	Gobiidae	*Vanneaugobius canariensis*
1	0.00	Actinopterygii	Lethrinidae	*Monotaxis grandoculis*
1	0.00	Actinopterygii	Psychrolutidae	*Cottunculus thomsonii*
1	0.00	Actinopterygii	Gobiidae	*Deltentosteus collonianus*
1	0.00	Actinopterygii	unclassified	unclassified
1	0.00	Elasmobranchii	Myliobatidae	*Rhinoptera bonasus*
1	0.00	Actinopterygii	Centracanthidae	*Spicara maena*
1	0.00	Actinopterygii	Centrolophidae	*Centrolophus niger*
1	0.00	Actinopterygii	Gobiidae	*Millerigobius macrocephalus*
1	0.00	Actinopterygii	Myctophidae	*Myctophum asperum*
1	0.00	Actinopterygii	Balistidae	unclassified
1	0.00	Elasmobranchii	Carcharhinidae	unclassified
1	0.00	Actinopterygii	Gobiidae	*Pomatoschistus knerii*
1	0.00	Actinopterygii	Soleidae	*Pegusa lascaris*
1	0.00	Actinopterygii	Anguillidae	*Anguilla anguilla*
1	0.00	Actinopterygii	Moridae	*Halargyreus johnsonii*
1	0.00	Actinopterygii	Myctophidae	*Lampadena atlantica*
1	0.00	Actinopterygii	Gobiidae	*Gobius cobitis*
1	0.00	Actinopterygii	Cyprinodontidae	unclassified
1	0.00	Actinopterygii	Belonidae	*Tylosurus crocodilus*
1	0.00	Actinopterygii	Gobiidae	*Periophthalmus barbarus*
1	0.00	Actinopterygii	Myrocongridae	*Myroconger compressus*
1	0.00	Actinopterygii	Gigantactinidae	*Gigantactis vanhoeffeni*
1	0.00	Actinopterygii	unclassified	unclassified
1	0.00	Actinopterygii	Cyprinidae	*Alburnus alburnus*
1	0.00	Actinopterygii	Nettastomatidae	*Venefica proboscidea*
1	0.00	Actinopterygii	Pleuronectidae	unclassified
1	0.00	Actinopterygii	Lotidae	*Molva dypterygia*
1	0.00	Actinopterygii	unclassified	unclassified
1	0.00	Actinopterygii	Myctophidae	*Myctophum nitidulum*
1	0.00	Actinopterygii	Notacanthidae	*Polyacanthonotus rissoanus*
1	0.00	Actinopterygii	Gasterosteidae	unclassified
1	0.00	Actinopterygii	Pleuronectidae	*Platichthys flesus*
1	0.00	Actinopterygii	Chiasmodontidae	*Dysalotus alcocki*
1	0.00	Actinopterygii	Macrouridae	*Trachonurus sulcatus*
1	0.00	Actinopterygii	Clupeidae	*Alosa pseudoharengus*
1	0.00	Actinopterygii	Carangidae	*Naucrates ductor*
1	0.00	Actinopterygii	Anotopteridae	*Anotopterus pharao*
1	0.00	Actinopterygii	Gobiidae	unclassified
1	0.00	Actinopterygii	Cyprinidae	*Alburnus chalcoides*

**FIGURE 2:**
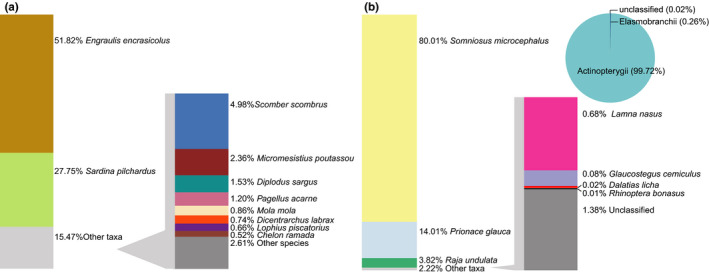
Relative number of “teleo” reads (%) assigned to (a) Actinopterygii and (b) Elasmobranchii species recovered from eDNA metabarcoding. Note that 4.96% Actinopterygii were not classified into species level

More than half of the reads are assigned to European anchovy, *E. encrasicolus* (51.67%), followed by European pilchard, *S. pilchardus* (27.67%), Atlantic mackerel, *Scomber scombrus* (4.96%), blue whiting, *Micromesistius poutassou* (2.36%), white seabream, *Diplodus sargus* (1.52%), and axillary seabream *Pagellus acarne* (1.20%), which together represent 89.38% of the reads (Figure 2a). A small percentage of the reads (0.27%) were classified as Elasmobranchii, including seven species such as the Greenland shark, *Somniosus microcephalus*, the blue shark, *Prionacea glauca*, and the undulate ray, *Raja undulata* (Figure 2b). The remaining reads were assigned to species that represent each less than 1% of the total number or reads.

**FIGURE 3:**
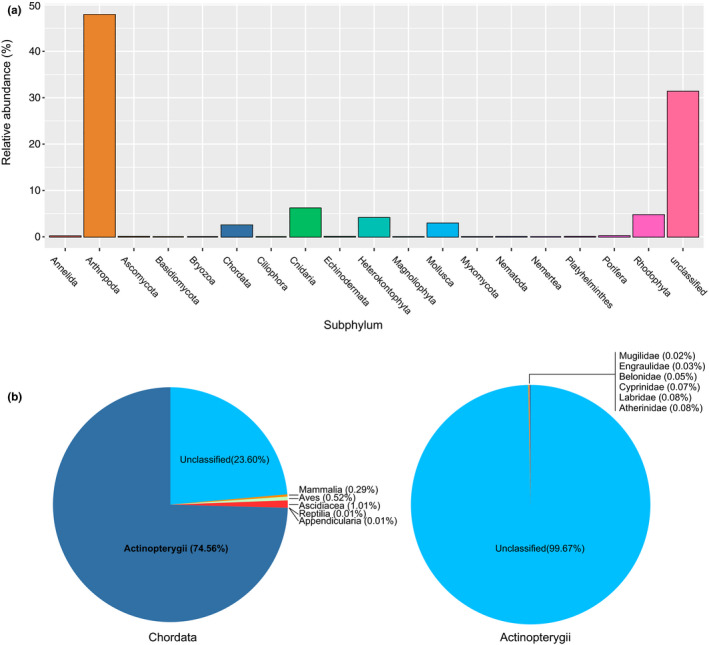
(a) Relative read abundance (%) of taxa classified to Subphylum, and (b) specifically classes within Chordata and families within Actinopterygii, respectively, from the four samples sequenced with the “mlCOI” primers

As for the four samples amplified with “mlCOI” primers, we obtained 389,665 raw reads from which, 324,731 (83%) were retained for downstream analyses. The average number of “mlCOI” reads per sample retained after quality filtering is 81,183 (Table 3). Using the BOLD database, 89.86% of the reads were classified into Phylum, 80.87% of which were metazoans, and among them 47.88% were classified as arthropods and 2.51% as chordates (Figure 3). Within chordates, 74.56% of the reads were classified as Actinopterygii (1.87% of the overall reads), resulting in only seven taxa classified into species (Figure 3).

**TABLE 3:** Station depth, category, and number of reads obtained per sample after sequencing, removing primers, pair‐assembling, quality filtering, primer mapping, and chimera removal for the *mlCOI* region

Sample	Station depth (m)	Category	Raw	Retained after primer checking	Retained after merging	Retained after quality filtering	Retained after mapping to coi region	Retained after chimera removal	% of retained reads for analysis
Sample_01	27	Shallow	103,773	103,171	102,988	102,988	86,259	82,595	79.59
Sample_27	1,003	Deep	98,307	97,931	97,886	97,886	86,798	82,874	84.30
Sample_32	185	Deep	98,873	98,095	98,013	98,013	87,716	83,993	84.95
Sample_47	675	Deep	88,712	88,348	88,294	88,294	78,173	75,269	84.85
TOTAL			389,665	387,545	387,181	387,181	338,946	324,731	
AVERAGE_all			97,416.25	96,886.25	96,795.25	96,795.25	84,736.50	81,182.75	83.42

### 3.2 Comparison with fish trawling

**FIGURE 4:**
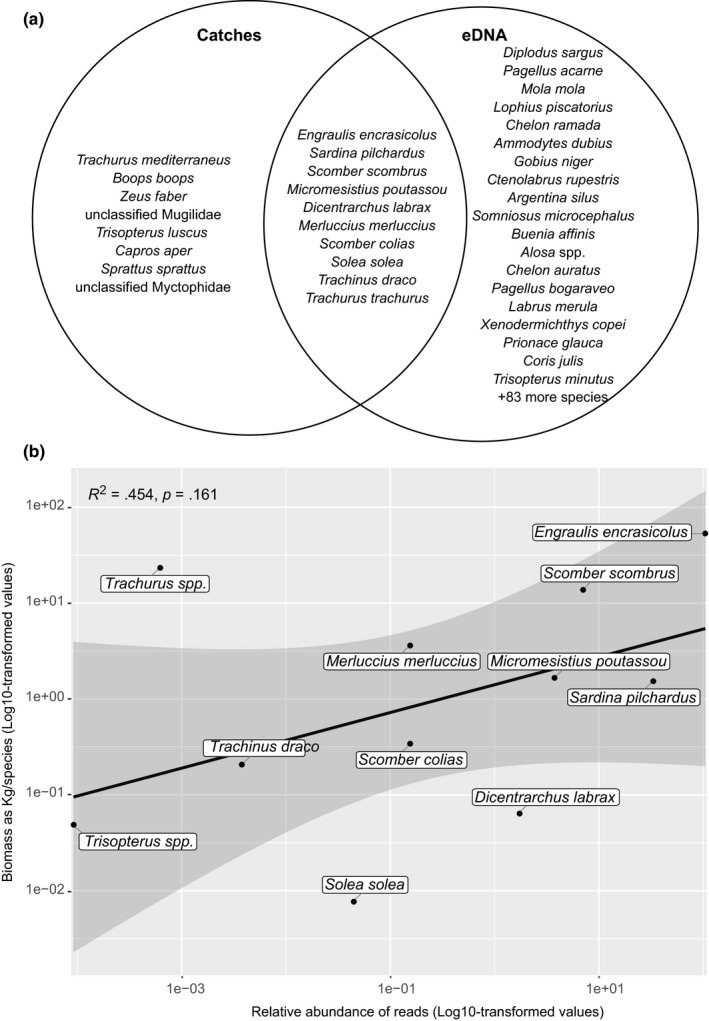
(a) Venn diagram showing fish species caught in trawls and detected through eDNA metabarcoding organized in decreasing order according to biomass or number of reads. (b) Relationship between the log10‐transformed values for the number of reads and biomass in kg from all fish species simultaneously found through eDNA and caught during fish trawling. Shaded area represents the 95% confidence interval of the linear regression

**FIGURE 5:**
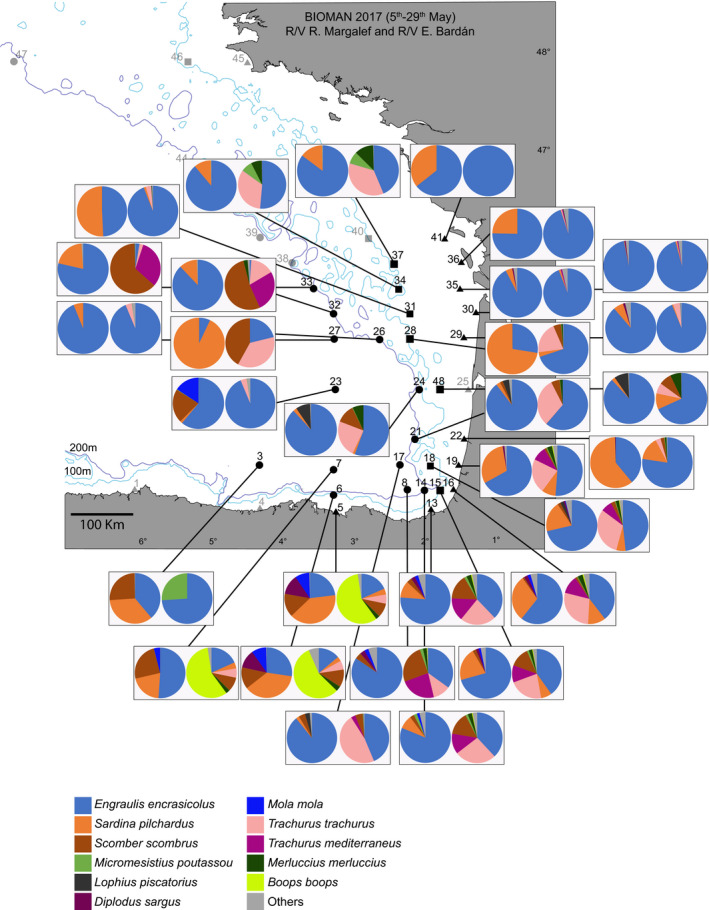
Pie charts showing the relative abundance of eDNA reads (first chart) and fish biomass caught (second chart) obtained from the 30 groups of stations within a 20 nm ratio. eDNA charts include species with >10 reads only. Species with >5% biomass caught/number of reads per station are coded by colors, the rest are grouped in “others”

Trawling operations during the BIOMAN survey resulted in a total of 18 taxa caught, from which lanternfishes (Fam. Myctophidae) and mullets (*Mugil* sp.) were the only ones not classified into species level. Qualitatively, a total of 10 species were identified both from the eDNA and trawling catches (Figure 4a) and even considering only the overlapping region between both sampling methods, eDNA resulted in 102 more species than catches. Six species were collected during catches and not detected through eDNA, namely *Sprattus sprattus*, *Trachurus mediterraneus*, *Boops boops*, *Zeus faber*, *Trisopterus luscus*, and *Capros aper* (Table 4); from these, there are no sequences for *T. mediterraneus* and *B. boops* in the reference database and the fact that we find *T. minutus* in eDNA suggest that this could be actually *T. luscus*. To assess the relationship between the biomass of fish caught and the number of reads obtained through eDNA, data from *T. mediterraneus* and *T. trachurus* were combined into *Trachurus* spp. and that from *T. luscus* and *T. minutus* into *Trisopterus* spp. There was an overall correlation between fish biomass and number of reads per species although not significantly different from 0 at *p* < .05 (Figure 4b). *E. encrasicolus* was the most abundant species for both methods, while the relative abundance for some species like *Dicentrarchus labrax*, *M. poutassou*, and *S. pilchardus* was higher when using eDNA. In contrast, the relative abundance of *M. merluccius*, *S. scombrus*, and *Trachurus* spp. was higher in catches than when using eDNA (Figure 4b; Table 4). At a local scale, no significant correlation between eDNA and trawling‐based abundances was found (Mantel test, *r* = −0.04 *p* = .646). In fact, eDNA data showed a more constant abundance of the three most abundant species (*E. encrasicolus*, *S. pilchardus*, and *S. scombrus*), compared to trawl data, which showed in general a higher number of species per station, except for those eight stations were *E. encrasicolus* was dominant (>94% of the catch) (Figure 5).

**TABLE 4:** Biomass (Kg/species) caught in fishing trawls compared with the number of reads obtained through eDNA. The total number of reads does not include sites north La Rochelle

Species	Number of reads	%	Biomass (kg)	%
*Boops boops*	0	0.00	8.26	1.10
*Capros aper*	0	0.00	0.34	0.05
*Dicentrarchus labrax*	13,712	0.45	0.36	0.05
*Engraulis encrasicolus*	1,722,690	56.94	400.33	53.31
*Merluccius merluccius*	4,454	0.15	27.49	3.66
*Micromesistius poutassou*	81,649	2.70	12.44	1.66
*Mugil* sp.	—	—	0.90	0.12
Myctophidae	—	—	0.27	0.04
*Sardina pilchardus*	621,400	20.54	11.49	1.53
*Scomber colias*	4,464	0.15	2.57	0.34
*Scomber scombrus*	149,397	4.94	104.86	13.96
*Solea solea*	591	0.02	0.05	0.01
*Sprattus sprattus*	0	0.00	1.07	0.14
*Trachinus draco*	151	0.00	1.56	0.21
*Trachurus mediterraneus*	0	0.00	49.59	6.60
*Trachurus trachurus*	29	0.00	126.98	16.91
*Trisopterus luscus*	0	0.00	0.36	0.05
*Trisopterus minutus*	5	0.00	0.00	0.00
*Zeus faber*	0	0.00	2.07	0.28

### 3.3 Species distribution patterns

**FIGURE 6:**
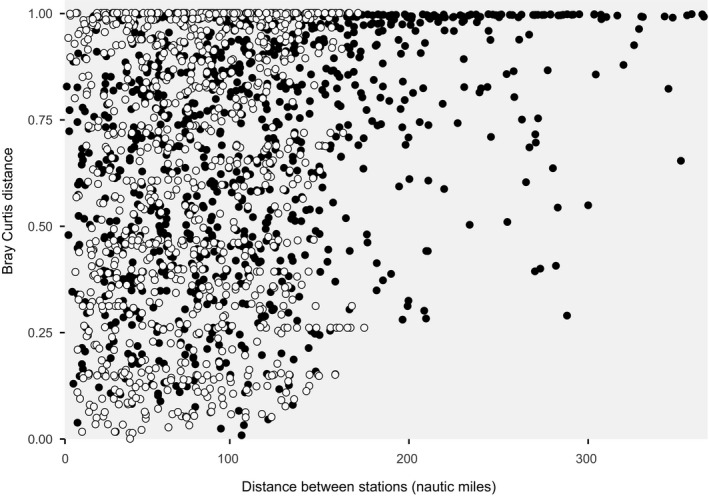
Scatterplot showing the overall relationship between Bray–Curtis distance and geographic distance between pairs of eDNA (black) and trawling (white) stations

**FIGURE 7:**
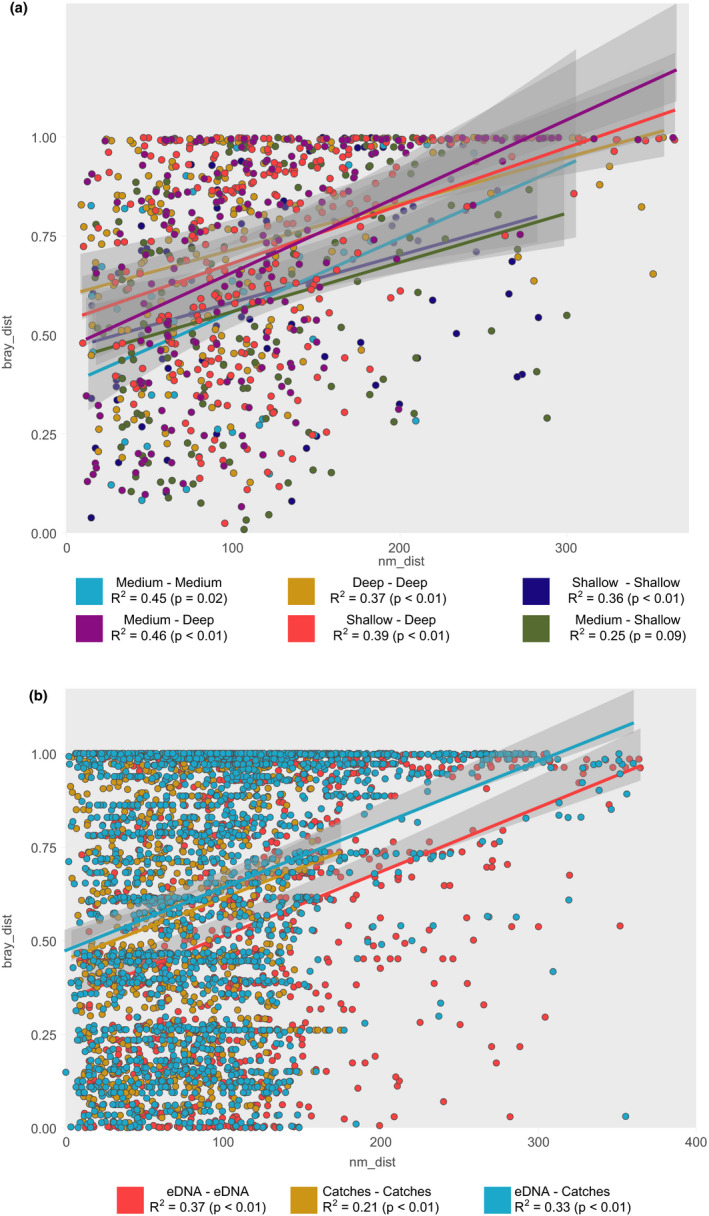
Scatterplot showing the relationship between Bray–Curtis distance and geographic distance between pairs of sampling points for (a) eDNA, (b) trawling, and (c) eDNA and trawling stations combined. Species included in c are only the common species detected by the two sampling methods. Pearson correlation is shown for each data group. Shaded area represents the 95% confidence interval of the linear regression

We found that correlation between compositional dissimilarities and geographic distances between stations was weak for both eDNA (*R* ^2^ = .38 *p* < .01) and trawling stations (*R* ^2^ = .20 *p* < .01). In both cases, pairs of stations that are less than about 100 nautical miles apart cover the full range of Bray–Curtis distances (Figure 6), whereas more distant stations differ more in taxonomic composition. This is particularly evident for eDNA samples, for which pairs of stations that are more than 200 nautical miles apart are available. Comparisons between samples within same or distinct depth category (shallow, medium, deep) or within same or distinct sampling methods (eDNA, trawling) had no effect over the observed patterns (Figure 7).

**FIGURE 8:**
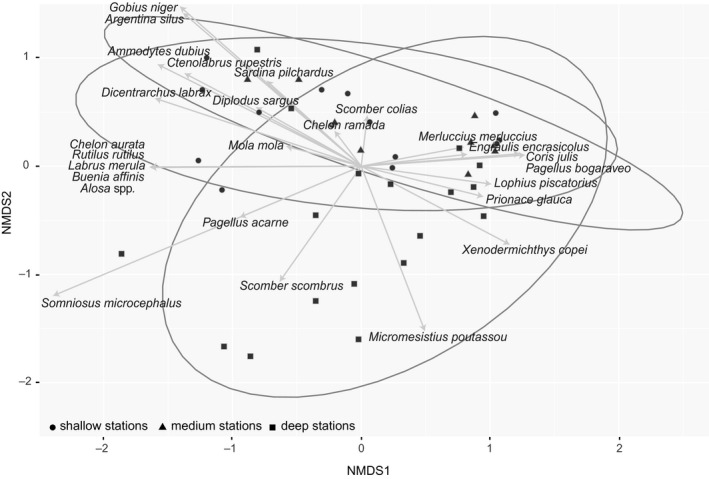
Nonmetric multidimensional scaling (NMDS) plot, with a stress of 0.15, showing the similarity of species from each sample based on their relative abundance. The ellipse shows the 95% distance based on the centroid of the three sampling zones groups (shallow, medium, and deep stations). Spatial patterns of the species with >1,000 reads are shown

**FIGURE 9:**
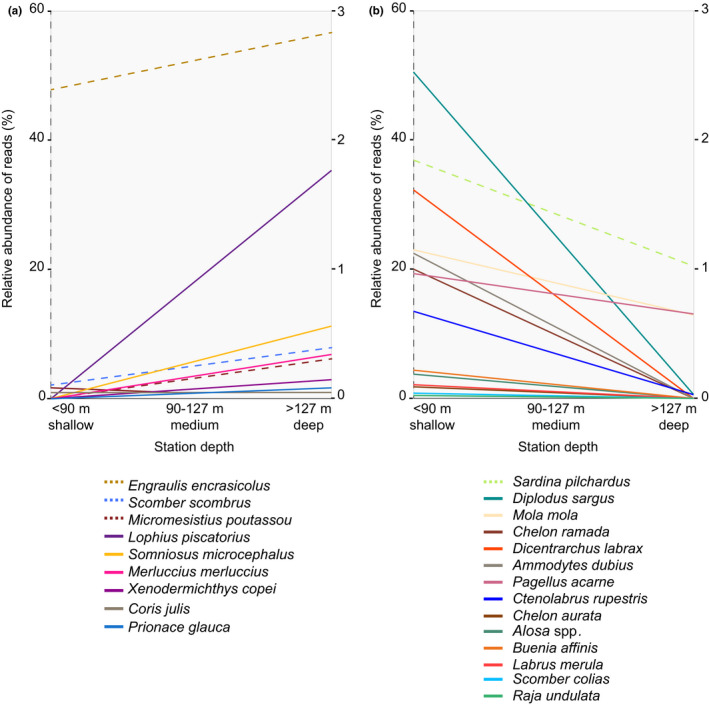
Linear relationship between depth and the relative abundance (in number of reads) obtained for those species with >1,000 reads, indicating those that increase (a) or decrease (b) with depth. For clarity, the more abundant species are represented with dashed lines on the left‐hand y‐axis, and the least abundant ones, with continuous lines to the right‐hand y‐axis

The overall compositional pattern of our data showed significant differences between species occurrence and sampling sites according to their zone (e.g., shallow, medium, and deep stations) (PERMANOVA *F*_2,43_ = 2.24, *p* < .05) (Figure 8). Within the main species contributing to the spatial ordination of our data, two main groups can be broadly observed. On one side, species like *E. encrasicolus*, *M. merluccius*, *Coris julis*, *S. scombrus*, *M. poutassou*, *Lophius piscatorius*, *S. microcephalus*, *Xenodermichthys copei*, and *P. glauca* tended to be more abundant in deeper stations and their relative abundances increased in sites > 127‐m deep (Figure 9). In contrast, a second loop in the spatial ordination of the data include other species such as *Gobius niger*, *Ammodytes dubius*, *D. sargus*, *Argentina silus*, *D. labrax*, *S. pilchardus*, *Mola mola*, and *Scomber colias* (Figure 8). This information correlates with a pattern of higher abundance in <90 m‐deep sites for, for example, *S. pilchardus*, *D. sargus*, *M. mola*, *A. dubius*, *D. labrax*, and *S. colias* (Figure 9). Relatively to the abundance of reads and station depth, four species, namely *A. silus*, *Glaucostegus cemiculus*, *G. niger*, and *Pagellus bogaraveo*, remain unchanged between shallow and deep stations. Specifically, for elasmobranch species, a pattern correlated with higher relative abundances of typical demersal species like *R. undulata* in shallow sites and pelagic species like *S. microcephalus* and *P. glauca* in medium and deep sites (Figure 9). Species like *Labrus merula* and *Buenia affinis* were among the most abundant in number of reads (>1,000 per species) but have not been previously reported for the Bay of Biscay.

## 4 DISCUSSION

This study shows how eDNA metabarcoding provides a comprehensive overview of the fish diversity in a large‐scale marine area. Compared to fish trawling, eDNA metabarcoding was able to “capture” a larger number of fish species. Both, eDNA and trawling‐based estimates (in number of reads and biomass, respectively) indicate that *E. encrasicolus* represents half of the abundance, which is consistent to the known large and stable anchovy population in the Bay of Biscay (Erauskin‐Extramiana et al., 2019; Santos, Uriarte, Boyra, & Ibaibarriaga, 2018; Uriarte, Prouzet, & Villamor, 1996) and with the fact that the BIOMAN survey took place during the anchovy spawning season. The seven most abundant species in fish trawling representing > 1% of the total biomass were *T. trachurus*, *S. scombrus*, *T. mediterraneus*, *M. merluccius*, *M. poutassou*, *S. pilchardus*, and *B. boops*, which were all, except those not present in the reference database (*B. boops* and *T. mediterraneus*), also found in the eDNA metabarcoding data, and four of them (*E. encrasicolus*, *S. pilchardus*, *S. scombrus*, and *M. poutassou*) were also among the most abundant species from eDNA data. Thus, concerning the most abundant species in the Bay of Biscay, eDNA and trawling data provided comparable conclusions.

**FIGURE 10:**
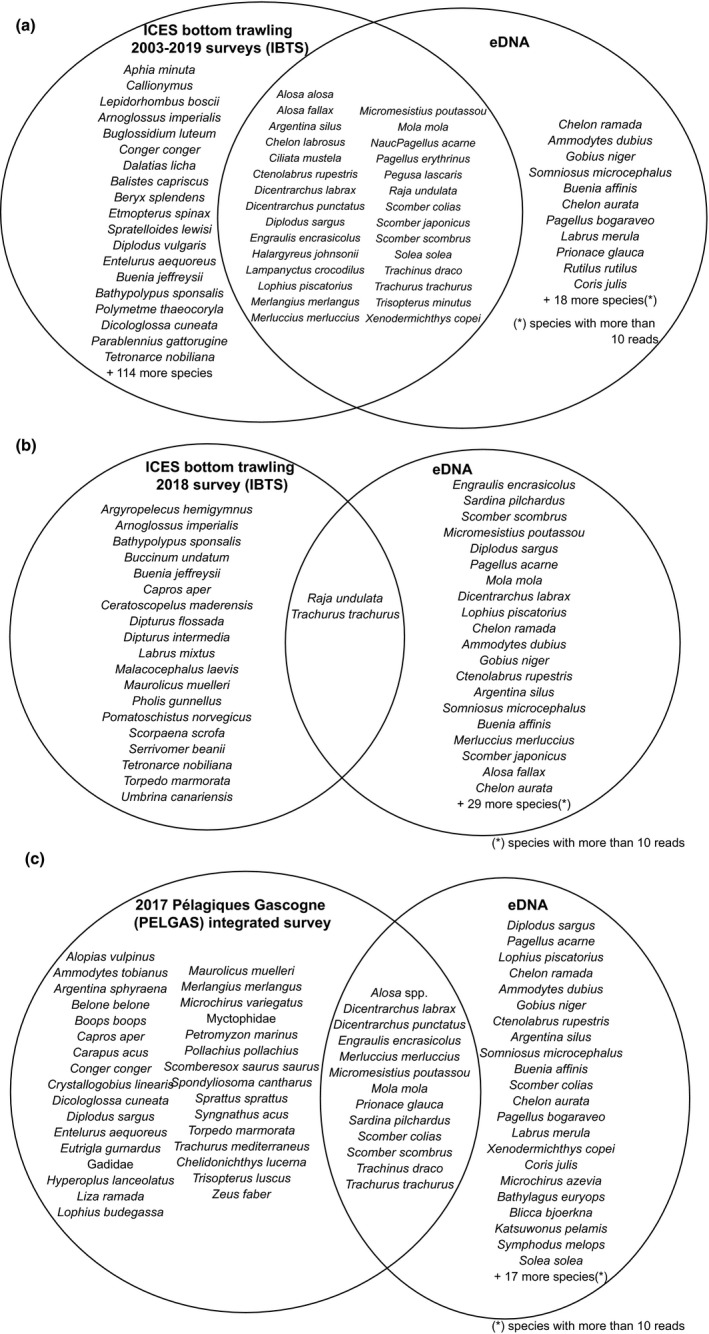
Venn diagrams showing fish caught in the ICES Bottom Trawling Survey carried out (a) between 2003 and 2019 and (b) in October 2018 available from ices.dk/marine‐data/data‐portals/ and (c) in the 2017 Pélagiques Gascogne (PELGAS) integrated survey compared to the fish species detected through eDNA metabarcoding

The following three species were caught during fish trawling but were absent from eDNA data despite being present in the reference database, *Z. faber*, *S. sprattus*, and *C. aper*. One possible explanation for this false‐negative detection could be the little abundance of this species’ DNA in the water, as suggested by the small and reduced number of catches (2.07 Kg in 3 sites, 1.07 kg in 2 sites, and 0.34 Kg in 2 sites, respectively). In fact, a small number of reads, that is, 591, was also detected for *Solea solea*, a species from which 0.05 kg were caught in a single station. If this is the case, filtering larger volumes of water and increasing sequencing depth could improve detection. Alternatively, reference sequences for *Z. faber*, *S. sprattus*, and *C. aper* could be undetected errors in the reference database (Li et al., 2018) or correspond to alternative intraspecific variants. On the other hand, in accordance with previous studies, eDNA data resulted in about 100 more species (35 with more than 10 reads) than trawling data collected simultaneously (Thomsen et al., 2012, 2016; Yamamoto et al., 2017). For example, species such as *D. sargus*, *P. acarne*, *M. mola*, *D. labrax*, *L. piscatorius*, *Chelon ramada*, *A. dubius*, *G. niger*, *Ctenolabrus ruperstris*, *A. silus*, *S. microcephalus*, *and B. affinis* were not found in catches, but were more abundant in eDNA reads than the 5th most abundant species (*M. merluccius*) in catches. The fact that eDNA results in a higher number of species could be partially attributed to the efficiency of the method to detect benthic or coastal species, difficult to catch by pelagic trawling nets, focused on small and medium‐size pelagic species. To check to what extent eDNA is able to detect in surface waters (4 m) demersal species, we compared the results with the ICES International Bottom Trawling Surveys (IBTS surveys) data for the Bay of Biscay from 2003 to 2019 (ICES, 2013) and with the 2017 Pélagiques Gascogne (PELGAS) integrated survey in the same area (Mathieu et al., 2019). eDNA metabarcoding data were able to detect at least 31 out of 164 species reported for the Bay of Biscay by IBTS surveys and 13 out of 45 species by PELGAS survey (Figure 10). Yet, according to the bias‐corrected Chao II estimator, the species richness obtained from eDNA would be around 161, which is closer to the IBTS based estimation. Although not being a thorough comparison, as time periods and sampling seasons at least from IBTS surveys are different, the comparison provides an overall sense of eDNA as a potential method for surveying a large marine area in a relatively simple way. Differences in eDNA and pelagic trawl catchability can also explain the differences in relative abundances of the species found by the two kind of sampling methods, such as *S. pilchardus*, *M. poutassou*, and *D. labrax*, with higher number of eDNA reads relative to the biomass caught, or *T. trachurus*, *S. scombrus*, and *M. merluccius*, showing the opposite. However, similarity between both eDNA and trawling stations suggests that stations further apart tend to be more different. The amount, quality, and stability of DNA molecules are largely affected by the production rate from each organism, diffusion of the molecules in the water, and its inherent degradation (Barnes & Turner, 2016; Collins et al., 2018; Murakami et al., 2019; Thomsen et al., 2012). But also, PCR amplification stochasticity and sequencing depth are known to affect the number of reads obtained from an eDNA sample (DiBattista et al., 2017; Zinger et al., 2019).

*Trisopterus minutus*, a morphologically similar species to *T. luscus*, was identified through eDNA, which make us raise the hypothesis that specimens collected from catches were misidentified as *T. luscus*, potentially being *T. minutus* as eDNA revealed. This would not be an isolated case where morphological characteristics difficult to observe hamper taxonomic identification, and other available data (e.g., DNA) are needed for species identification (Dayrat, 2005). A remarkable case are lanternfishes of the Myctophidae, where species identification is based on the morphology and the shape and size of photophores, which are extremely fragile and seldom recovered intact (Cabrera‐Gil et al., 2018). In this case, eDNA can play a major role for species identification as this study has shown, where at least five myctophid species were identified through eDNA. On the other hand, erroneous database records or missing sequences can bias eDNA‐based estimates. The quality and completeness of the reference database is crucial for taxonomic classification of eDNA data (Callahan, McMurdie, & Holmes, 2017). For example, two species were among the most abundant in our dataset, but not reported previously in the Bay of Biscay, namely *L. merula* and *B. affinis*. A careful examination suggests that, although *L. merula* could be misled by its close relative *L. bimaculatus*, occurring in the Bay of Biscay, the sequences attributed to *B. affinis* seem to be correctly assigned, suggesting that eDNA was able to detect species not previously reported in the area despite in low abundance.

Besides species diversity, eDNA also provides information on species distribution, which is comparable to that expected in the area. For instance, the number of reads assigned to the pelagic species *M. poutassou* and *S. scombrus* increased in stations deeper than 90m, where preferred habitats for these species occur (Ibaibarriaga et al., 2007) even if samples were collected from the surface. A contrasting pattern was observed for the greater argentine *A. silus*, a species commonly found at depths between 50 and 200 m (Basterretxea et al., 2012), but found in our data at shallower stations. This could also suggest an incongruence with species identification with a close relative, in this case *A. sphyraena* commonly found over the continental slope (Basterretxea et al., 2012), but with no 12Sr RNA sequence in our reference database, or DNA from *A. silus* (even in its form of egg or larvae) dispersed to shallower stations. Similarly, species like *S. pilchardus*, *D. sargus*, *D. labrax*, *P. acarne*, and *Alosa* spp. showed a distribution for this dataset in stations less than 90m depth, as our eDNA revealed. Available data on the diversity of elasmobranch species in the Bay of Biscay are limited, as most of these species are discarded from commercial fisheries and landing data are incomplete (ICES, 2017; Rodríguez‐Cabello, Pérez, & Sánchez, 2013; Rusyaev & Orlov, 2013). Hence, in agreement to previous studies, our data support eDNA as a potential mechanism for detecting and studying the distribution of elusive and deep‐water species, which normally go undetected in fish trawl surveys, for example, elasmobranchs (Thomsen et al., 2016). In any case, eDNA results also revealed an ecological pattern for elasmobranchs, for instance *R. undulata*, which has a high‐site fidelity occurred only in shallow waters (ICES, 2014), while large sharks *as S. microcephalus*, *P. glauca* and *Lamna nasus* predominantly occurred in deeper sites. Interestingly, these differences were observed even when collecting water from the surface.

Aside from biological factors (e.g., individual shedding rate, persistence of DNA in the water) that can alter the quantity of eDNA released to the environment, technical considerations can introduce biases on the quality and number of reads generated per species and hence inferences driven from them (Dejean et al., 2011; Lamb et al., 2019; Thomsen et al., 2016). Reference databases are crucial to secure taxonomic assignment for data derived from eDNA samples (Zinger et al., 2019). While recent analyses on the taxonomic annotation of metazoan GenBank sequences suggest their reliability for eDNA metabarcoding studies (Leray, Knowlton, Ho, Nguyen, & Machida, 2019; Li et al., 2018), we encountered the need of including a thorough curation step for our “global” database giving several mislabeled sequences. Species‐level annotations were not considered in Leray et al. (2019), and we found incorrectly annotated sequences at all taxonomic levels. As environmental samples contain highly complex DNA signal from various organisms, primer choice is critical for species‐level identification (Collins et al., 2019). We found that for our samples, the eukaryote universal COI primers result in a very small proportion of reads assigned to Actinopterygii. This is due to the fact that the primers target a large number of taxonomic groups, so larger coverage is needed for producing robust data (Alberdi, Aizpurua, Gilbert, Bohmann, & Mahon, 2018; Corse et al., 2019; Gunther, Knebelsberger, Neumann, Laakmann, & Martinez Arbizu, 2018; Stat et al., 2017). The use of more specific primers in our study allowed the specific detection of both Actinopterygii and Elasmobranchii. (Kelly, Port, Yamahara, & Crowder, 2014; Miya et al., 2015). Yet the amount of reads attributed to Elasmobranchii is small as “teleo” primers were not specifically designed for this taxa, for example, Kelly et al. (2014), and recent developments on elasmobranch‐specific primers (Miya et al., 2015) could potentially be a powerful tool to increase the elasmobranch diversity in future marine surveys. In addition, for closely related species such as *Alosa alosa* and *Alosa fallax*, the target barcode was exactly the same, so being cautious we consider them as *Alosa* spp. Another crucial methodological step is the clustering method. We showed that using a clustering method (i.e., *vsearch* and *swarm*) decreased the number of identified species, probably because the algorithm merged similar sequences from different species into singular OTUs. Recent studies have suggested that clustering techniques and the use of percentages of similarities specially in short (<100 bp) sequences might mislead diversity estimates (Calderón‐Sanou, Münkemüller, Boyer, Zinger, & Thuiller, 2019; Callahan et al., 2017; Xiong & Zhan, 2018). Thus, procuring a taxonomically comprehensive database with good quality sequences and accurate data curation steps is crucial for producing robust and reproducible ecological conclusions from eDNA metabarcoding methods (Collins et al., 2019; Weigand et al., 2019). Including a human‐specific blocking primer in our samples had little effect, as we indeed detect, although a small percentage (<0.01%), reads identified as *H. sapiens*. The use of blocking primers in metabarcoding analysis has been previously used to block dominant taxa in a specific samples, for instance host DNA from diet analysis (Jakubavičiūtė, Bergström, Eklöf, Haenel, & Bourlat, 2017), or human DNA from ancient samples (Boessenkool et al., 2012). Our results suggest that our samples held very little contamination from external sources such as human manipulation, air, or input from land.

Alternative ways to survey marine biodiversity and unbiased evaluations of the ecosystem components are needed as these provide the baseline for policy implementation in the context of global marine directives (e.g., Common Fisheries Policy or the Marine Strategy Framework Directive). eDNA metabarcoding is becoming a more accessible method that generates reliable information for ecosystem surveillance and invites its application on regular marine monitoring programs (Bohmann et al., 2014; Lacoursière‐Roussel, Rosabal, & Bernatchez, 2016; Takahara, Minamoto, Yamanaka, Doi, & Zi, 2012). However, there is still discussion on whether eDNA‐based approaches can be used to manage fisheries, and there is a demand of continuous research to build confidence in eDNA‐based results as evidence (Jerde, 2019). This study has shown that eDNA samples provide information on fish diversity in a broad‐scale marine area such as the Bay of Biscay, detecting almost ten times more fish species compared with pelagic trawling, including some considered elusive or difficult to capture with traditional fishing methods. These results show that, despite its inherent uncertainties, eDNA metabarcoding has the potential to become a routine technique for fisheries management as it can provide information on fish diversity and distribution in large oceanic areas, including less accessible locations and targeting rare and elusive species, in a cost‐effective and noninvasive manner. This is particularly relevant in a context of global change, where establishing efficient management actions based on numerous, continuous, and accurate biodiversity assessments is paramount.

## CONFLICT OF INTEREST

The authors declare no conflict of interest.

## AUTHOR CONTRIBUTIONS

**Natalia Fraija‐Fernández:** Conceptualization (lead); Formal analysis (lead); Methodology (equal); Writing‐original draft (equal); Writing‐review & editing (equal). **Marie‐Catherine Bouquieaux:** Formal analysis (lead); Methodology (supporting); Writing‐review & editing (equal). **Anaïs Rey:** Formal analysis (supporting); Methodology (supporting); Supervision (supporting); Writing‐review & editing (equal). **Iñaki Mendibil:** Methodology (lead). **Unai Cotano:** Conceptualization (supporting); Funding acquisition (supporting); Resources (supporting); Writing‐review & editing (equal). **Xabier Irigoien:** Conceptualization (supporting); Funding acquisition (supporting); Resources (supporting); Writing‐review & editing (equal). **María Santos:** Conceptualization (supporting); Funding acquisition (supporting); Methodology (supporting); Resources (supporting); Writing‐original draft (supporting); Writing‐review & editing (equal). **Naiara Rodríguez‐Ezpeleta:** Conceptualization (lead); Formal analysis (equal); Methodology (lead); Project administration (lead); Resources (lead); Supervision (lead); Writing‐original draft (equal); Writing‐review & editing (equal).

## Data Availability

Raw sequencing reads are available at the NCBI SRA under Biosample accession numbers SAMN13489000‐SAMN13489051. Local database and scripts used for the preprocessing, clustering, and taxonomic assignment are available at https://github.com/rodriguez‐ezpeleta/fish_eDNAm.
